# A computerized expert system for diagnosing primary headache based on International Classification of Headache Disorder (ICHD-II)

**DOI:** 10.1186/2193-1801-2-199

**Published:** 2013-04-30

**Authors:** Vahid Eslami, Sadreddin Rouhani-Esfahani, Nima Hafezi-Nejad, Farshid Refaeian, Siamak Abdi, Mansoureh Togha

**Affiliations:** 1- Neurology ward, Sina Hospital, Tehran University of Medical Sciences, Tehran, Iran; Neurology Department, AJA University of Medical Sciences, Tehran, Iran; Iranian Center of Neurological Researches, Tehran, Iran; Foolad Institute of Technology, Foolad-Shahr, Iran; Shariati Hospital, Tehran University of Medical Sciences, Tehran, Iran

**Keywords:** Headache diagnosis, Software, International classification of headache disorder (ICHD-II)

## Abstract

**Background:**

The authors developed a computerized program designed to diagnose primary headache based on international classification of headache disorders, 2nd edition (ICHD-II) criteria for use by physicians.

**Methods:**

An appropriate questionnaire was designed according to the ICHD-II criteria for all types of primary headaches and the computerized system provided diagnosis based on the criteria. The software was tested by analyzing 80 patients, recruited from an outpatient headache clinic, affected by primary headache. Each patient with a unique card number was interviewed up to 15 minutes. At the end of each day, software and neurologist diagnoses were evaluated for each patient.

**Results:**

Of 80 patients, the software was able to come up with correct results in 78 cases. The age of the patients ranged from 30 to 80 years old. Migraine headache accounted for 71 cases, five patients had tension type headache, and 2 had cluster headaches; all were correctly diagnosed by software. Two cases were not concordant with the neurologist’s diagnosis. The neurologist diagnosed these two cases as “Medical overuse syndrome headache” and “cluster headache”, which our software was not able to diagnosis them.

**Conclusions:**

This software permitted the diagnosis of more than 97% of the patients similar to the physician's. We hope this questionnaire and applying the software to diagnose headache based on ICHD could be of help to better the diagnosis of headaches.

**Electronic supplementary material:**

The online version of this article (doi:10.1186/2193-1801-2-199) contains supplementary material, which is available to authorized users.

## Introduction

Headache is one of the most common complaints during life (Stang & Osterhaus 1993). During one year, 90% of people suffer from headaches, and over 10% have at least one migraine headache (Schwartz et al. 1998).

Although considerable advances are seen in the therapy of headache, it still remains underdiagnosed (Lipton et al. 2001). In the clinical setting, there are no possible imaging or laboratory tools to diagnose different types of primary headaches. However, the recently revised diagnostic criteria of International Classification of Headache Disorders, 2nd edition (ICHD-II) are the best available criteria to precisely diagnose the type of headache (2nd Edition of The International Headache Classification (ICHD-2) 2004. However, it is complicated for practitioners to memorize all ICHD-II criteria for the diagnosis of headaches which is about 150 pages long. On the other hand, the workload of practitioners obliges them to minimize the length of visits, therefore, making it often impossible to take a complete history and diagnose the headache as it should be in which case as a result, limits the quality of therapeutic choices.

For this reason, we developed a computerized program designed for use by physicians in populated headache clinics. This easy-to-use system provides an assisted diagnosis according to ICHD-II criteria for all types of primary headaches including migraine, tension-type headache, cluster headache and other trigeminal autonomic cephalalgias, as well as other primary headaches which are overall 61 types. Similar to other computerized programs (De Simone et al. 2007), this software can be used by operators with basic computer experience without increasing the time length of visits.

## Method

a. Software preparation

The software has 5 main parts including the descriptive page, disclaimer page, patient’s registration page, headache questions, and the final decision with the explanation of it, which are all prepared in Persian language (Additional file 1). The disclaimer page emphasizes that this software is made to facilitate and increase the accuracy of diagnosis.

The criteria were extracted from primary headaches. Then, based on these criteria, questions were designed to approach all these criteria. Questions would be asked based on responses, which take between 5 to 15 minutes depending on the answers. For example, if the patient has visual problem during a headache attack, further questions such as the association of headache with scotoma, flickering, scintillation, visual loss, diplopia, and bilateral/unilateral visual disturbances, and also the maximum duration of these symptoms would be asked. The system will get into conclusion based on all criteria of ICHD-II and explain how headache was approached and possible diagnosis was made.

An essential feature of the program is to report “No diagnosis was made based on these data”, which can also find certain unclassifiable headaches.

Furthermore, all demographic information and characteristics of the patients' headache, previously inserted in the program, are saved, allowing the creation of a complete database in Access software which would be an invaluable tool for more headache researches such as further modifications of ICHD-II.

Another feature of this software is that each patient has a profile, which can be updated at each successive follow-up. The profile is accessible to record the diagnosis, the recommended therapy and any further comments. Those who help the patient to answer the questions should not necessarily know the whole terminology of the words, since each terminology is simply explained by informative notes and explanations indicated by moving the pointer of the mouse over it.

b. Evaluating the software

We conducted a validation field-test to approve the accuracy of the software. In all, 95 consecutive patients were enrolled in our study from October to December 2012. Of them, 80 patients with primary headache were evaluated. The patients were recruited from an outpatient headache clinic, affiliated to Tehran University of Medical Sciences. By entering the study, each patient received a card with a unique number on it. The patients were primarily interviewed by the researchers. Each interview lasted up to 15 minutes. The data were collected from patients to fulfill the software’s entry. The questions were instructed based on ICHD-II. The software output described the proposed diagnosis. The software’s diagnosis was recorded for the card number each patient had. Next, in the same visit, the patients were interviewed by a neurologist in another room. The neurologist and the researcher were not aware of each others’ diagnosis. The patient was not informed of the software output either. The neurologists’ final diagnosis was separately recorded for the card number each patient had. The neurologist was asked to define the final first-ranked diagnosis by choices in concordance with ICHD-II classification. At the end of each day, software and neurologist diagnoses were evaluated for each patient.

### Ethical approval

The approval of local research ethics committee of Tehran University of Medical Sciences and verbal consents from all patients were obtained before any evaluation. The patients were informed that no-participation would in no way affect their care.

## Result

A total of 80 patients, over 18 years of age, were included in the study. Female patients outnumbered male patients (female patients n = 62). The age of patients ranged from 30 to 80 years old. The software was able to come up with correct results in 78 out of 80 cases. Migraine headache accounted for 71 cases, five patients had tension type headache, and 2 had cluster headaches; all were correctly diagnosed by software. Two cases were not concordant with the neurologist’s diagnosis. The neurologist diagnosed these two cases as “Medical overuse syndrome headache” and “cluster headache”, which our software was not able to diagnosis them.

## Discussion

This computerized system uses simple human-like algorithmic logic to determine the most appropriate type of headache. However, similar to other medical software, the accuracy of diagnosis depends on the accuracy of patient responses. Also, the interviewer has an invaluable role to obtain accurate responses in comparison with a computer program.

In our evaluation, 2 patients had different diagnoses by the computer and the physician. The reason was inaccurate and different responses of these 2 patients to the interviewer and physician. Different computerized headache programs are designed for use by general practitioners (Mainardi et al. 2005) to neurologists (Sarchielli et al. 2005). Maizel et al. (2008), in their study using a computerized headache assessment tool, correctly identified 100% of the patients with episodic migraine and 85.7% with transformed migraine. Also, it correctly categorized all patients with tension-type headache, and cluster headache. Sarchielli et al. (2007), in their study using a computerized system, permitted correct diagnosis in 78% of 200 headache patients.

As these criteria are under change over time (Olesen 2011; Bigal et al. 2007; Olesen 2006), we hope that applying the software to diagnose headache based on ICHD could be of help for modifying the current diagnostic criteria, finding the answer to issues which need to be studied more in the ICHD-II guideline. However, in a clinical practice, we face a large number of patients who do not qualify for any defined criteria. As an example, a few criteria include a response to a particular drug. A marked overlap exists among criteria. On the other hand, the patients cannot fit their responses to the algorithm that any software undertakes. These all should be addressed before considering such type of design. Yet still, an expert neurologists’ diagnosis is the standard for comparing such software. For the same purpose, we conducted our validation survey in the neurologist’s clinic. However, this may result in further limitations. Many of the patients in such clinics are referred from other clinics due to the complicated nature of their headache or inconclusive diagnosis. They may have received different medications which might have altered the pattern of their headache, as well. For instance, one would have had one sided throbbing headache in the beginning, but recently has experienced both sided compression-like headache. Yet though, the software needs a definite input. We used the primary pattern of the headache as the input. Without the precise import of the specified inputs, the software would come up with a number of diagnoses including different types of headache. However, an expert neurologist can handle such situations through history taking and proper weighting of differential diagnoses. Also, patient’s willingness to cooperate and trust this method is another limitation to be mentioned. Finally, we recommend using such software to promote diagnosis in settings like general day clinics instead of sub-specialized neurology clinics.

## Electronic supplementary material

Additional file 1: **The mock-up translated screenshots of the software.** (DOC 1 MB)
