# Supplementary figures and images for: A computerized expert system for diagnosing primary headache based on International Classification of Headache Disorder (ICHD-II)
Source: Springerplus. 2013 Apr 30;2:199. doi: 10.1186/2193-1801-2-199 (PMC3661080; doi:10.1186/2193-1801-2-199)

Appendix 1. The mock-up translated screenshots of the software.


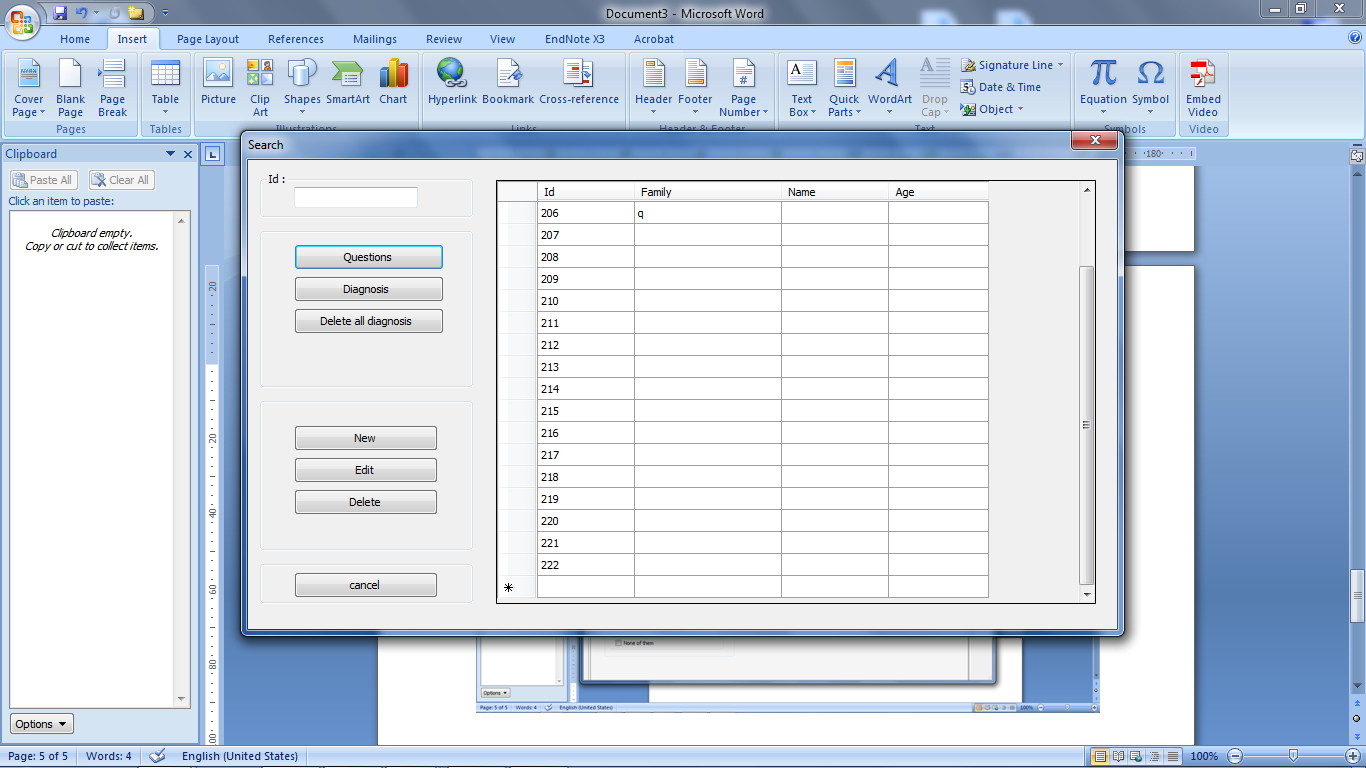


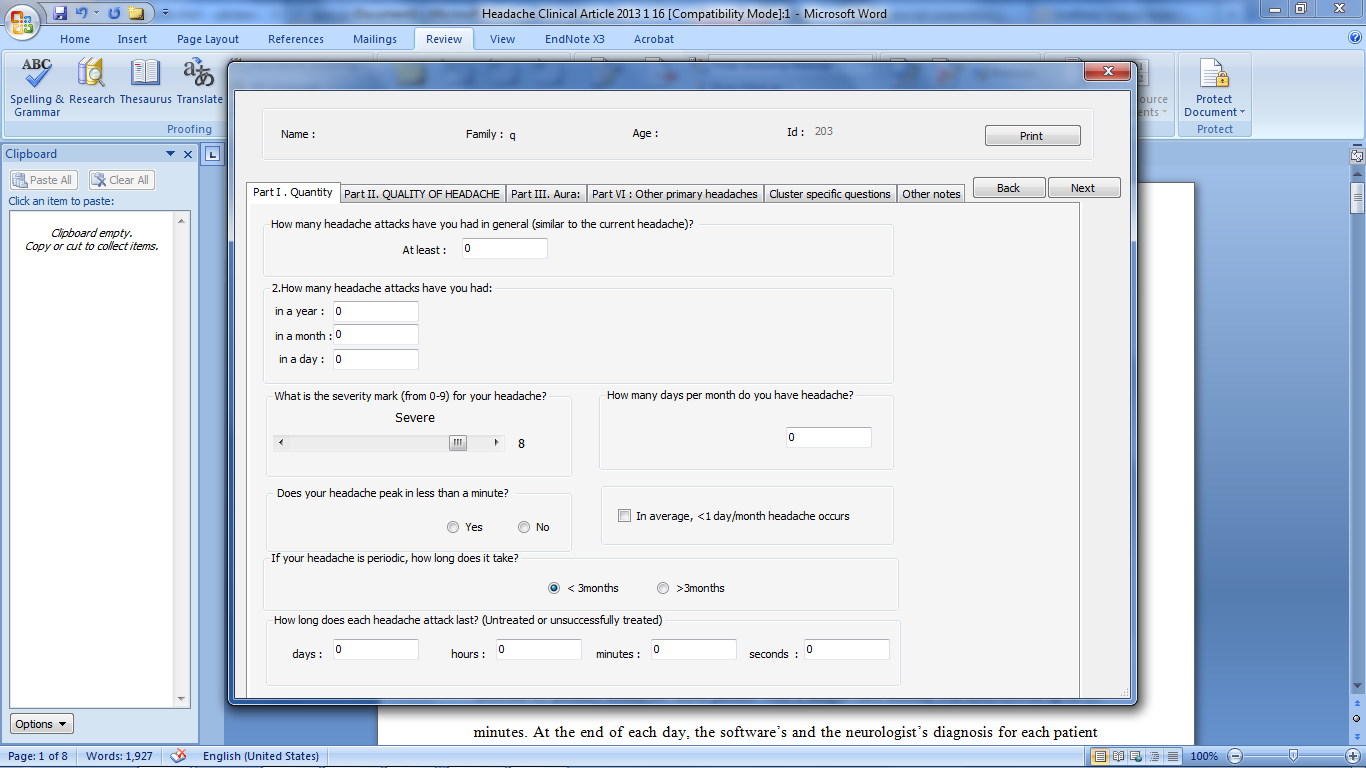


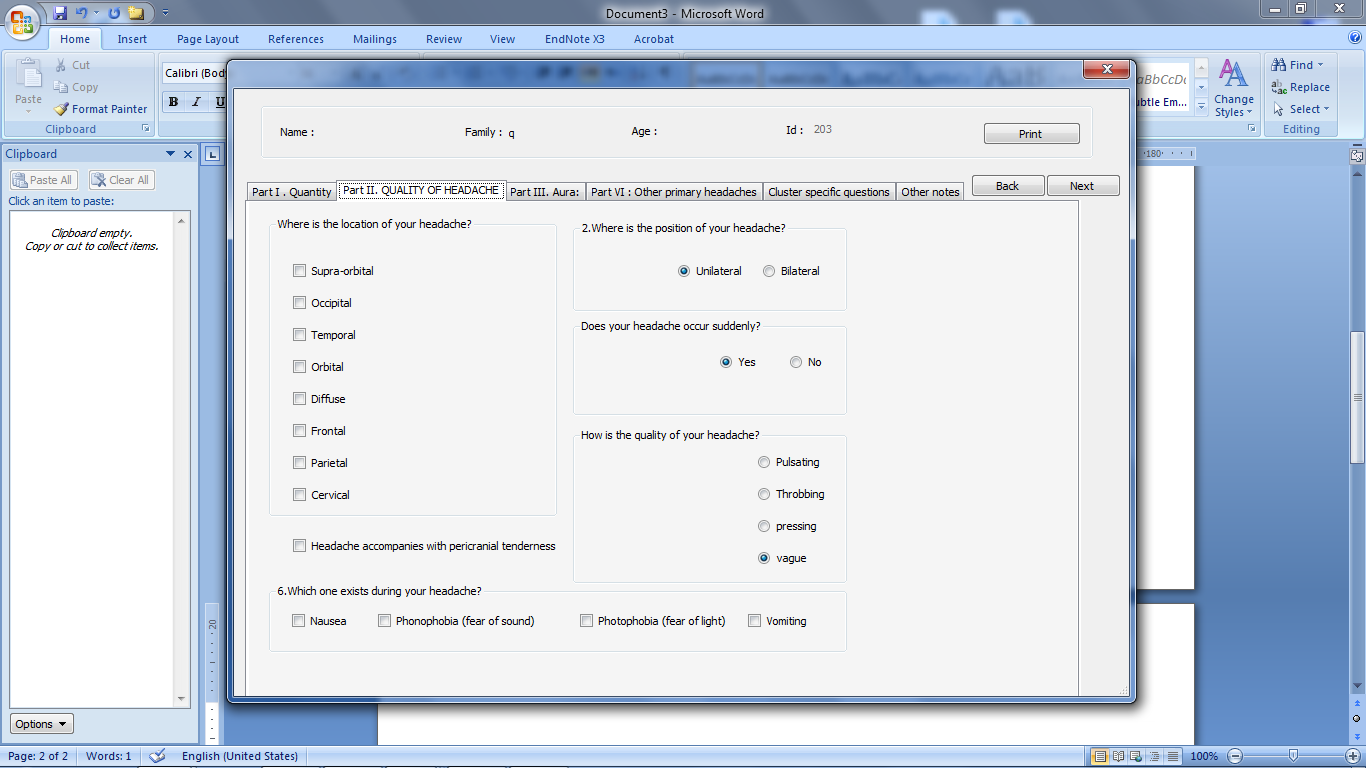


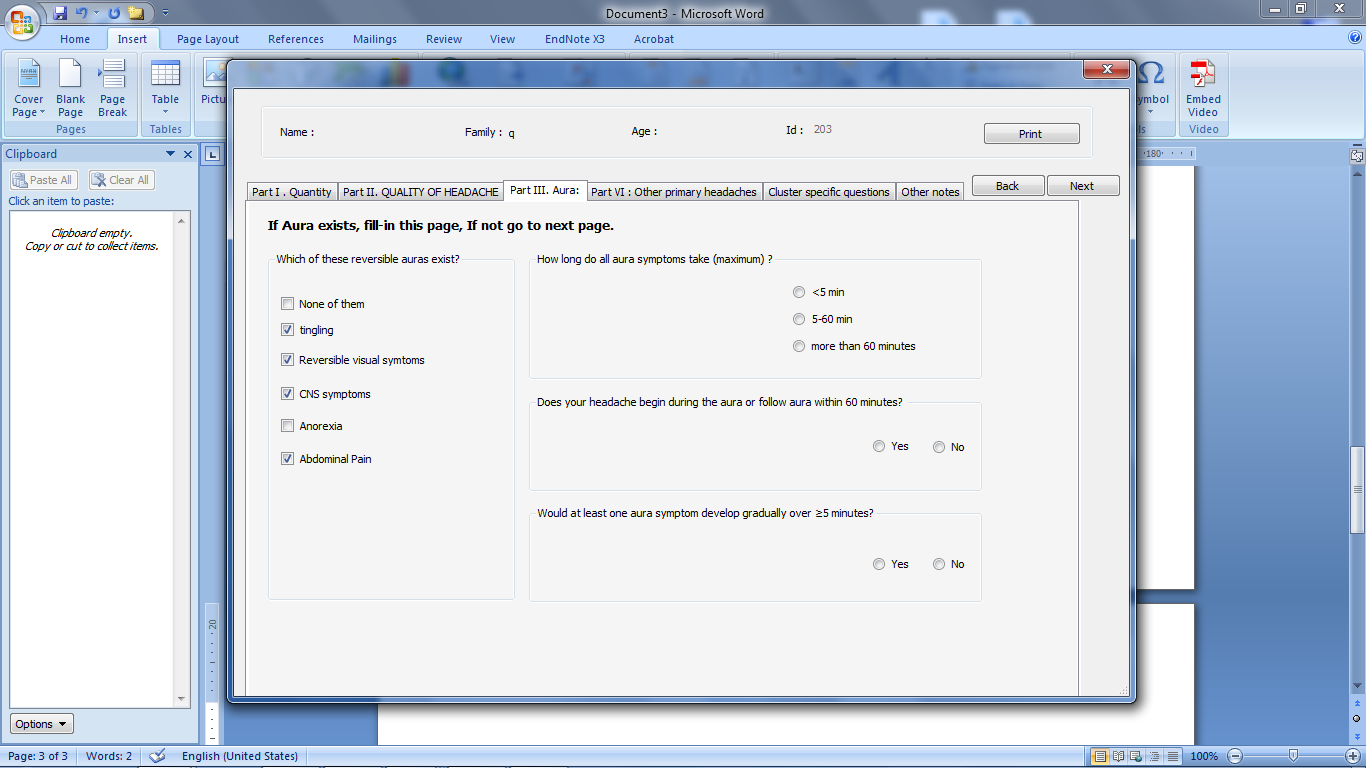


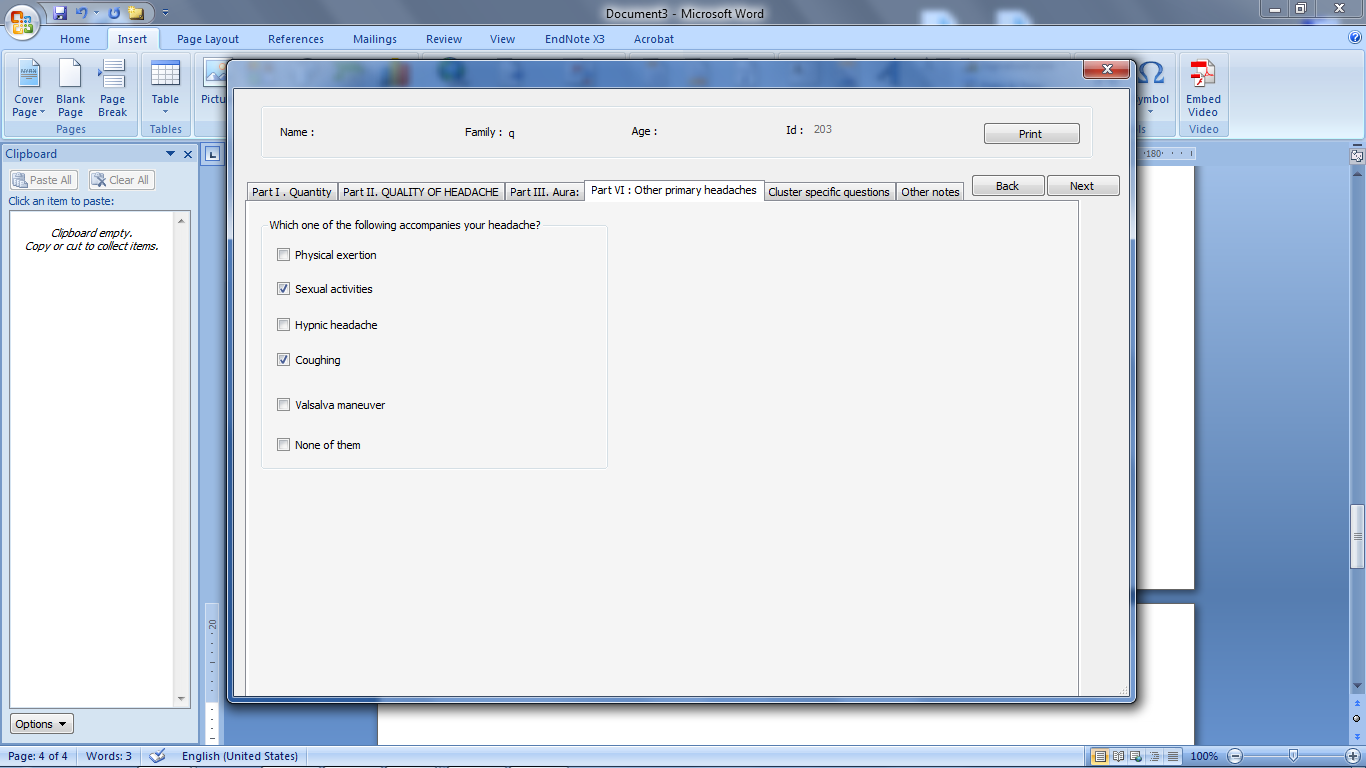


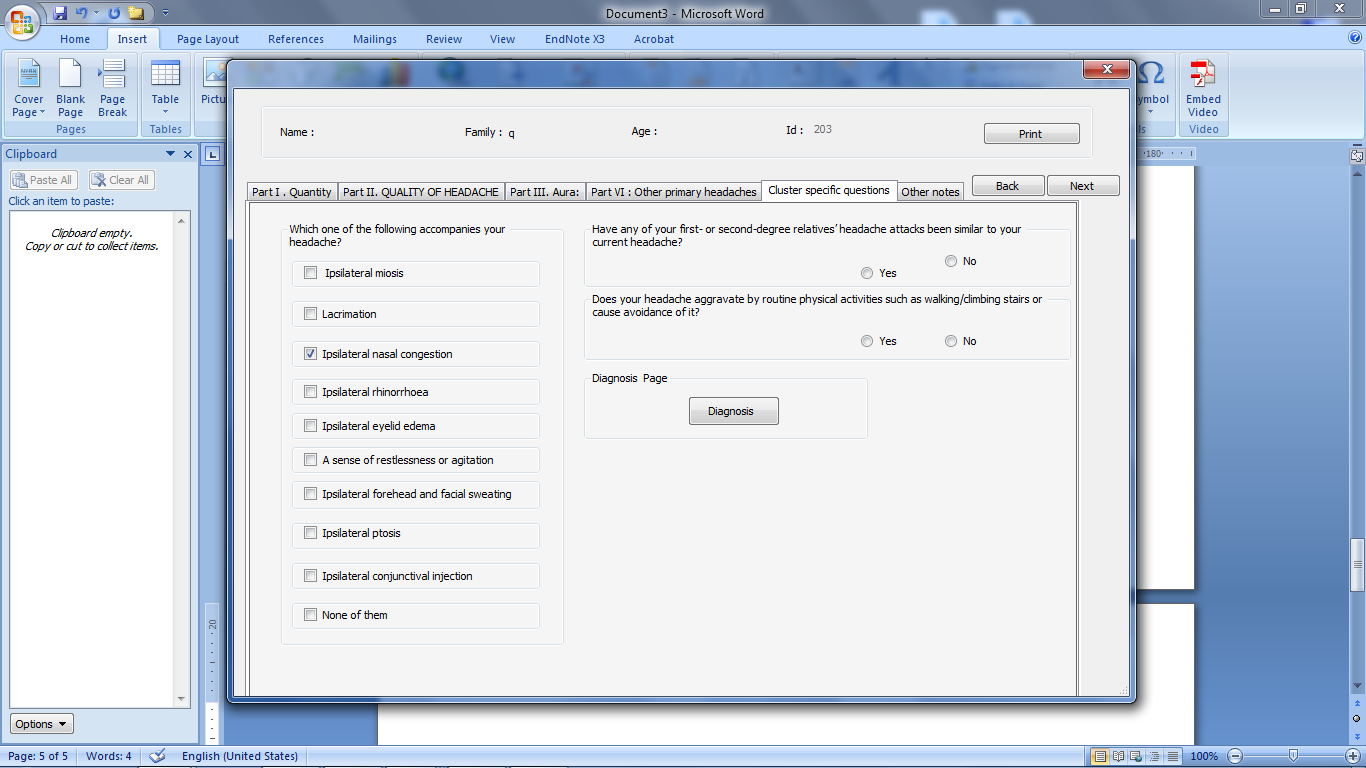

Supplement: Supplementary file 1 — Additional file 1: The mock-up translated screenshots of the software. (DOC 1 MB) [file 40064_2012_268_MOESM1_ESM.doc]
